# Framing international trade and chronic disease

**DOI:** 10.1186/1744-8603-7-21

**Published:** 2011-07-04

**Authors:** Ronald Labonté, Katia S Mohindra, Raphael Lencucha

**Affiliations:** 1 Institute of Population Health, University of Ottawa, Ottawa, Canada; 2 University of Lethbridge, Lethbridge, Alberta, Canada

## Abstract

There is an emerging evidence base that global trade is linked with the rise of chronic disease in many low and middle-income countries (LMICs). This linkage is associated, in part, with the global diffusion of unhealthy lifestyles and health damaging products posing a particular challenge to countries still facing high burdens of communicable disease. We developed a generic framework which depicts the determinants and pathways connecting global trade with chronic disease. We then applied this framework to three key risk factors for chronic disease: unhealthy diets, alcohol, and tobacco. This led to specific 'product pathways', which can be further refined and used by health policy-makers to engage with their country's trade policy-makers around health impacts of ongoing trade treaty negotiations, and by researchers to continue refining an evidence base on how global trade is affecting patterns of chronic disease. The prevention and treatment of chronic diseases is now rising on global policy agendas, highlighted by the UN Summit on Noncommunicable Diseases (September 2011). Briefs and declarations leading up to this Summit reference the role of globalization and trade in the spread of risk factors for these diseases, but emphasis is placed on interventions to change health behaviours and on voluntary corporate responsibility. The findings summarized in this article imply the need for a more concerted approach to regulate trade-related risk factors and thus more engagement between health and trade policy sectors within and between nations. An explicit recognition of the role of trade policies in the spread of noncommunicable disease risk factors should be a minimum outcome of the September 2011 Summit, with a commitment to ensure that future trade treaties do not increase such risks.

## Background

The nature and magnitude of the burden of chronic disease in low and middle-income countries (LMICs) is now well understood, as are its impacts on health systems and national economies [1-5]. What is less clear is how we should address chronic disease in LMICs, although doing so will require actions at both local and global levels [6]. At the global level, international trade, despite bringing potential health benefits through economic growth (a point we return to) is one of the major driving factors of a growing chronic disease burden. Trade's effects on chronic disease risk occur *progressively *along multiple pathways. It is the intent of this article to explicate those pathways, of particular importance given the high-level international attention now being directed to the global chronic disease burden.

Trade is not a new phenomenon: human societies have long histories of trade with each other and one might even describe barter and exchange as inherently human social qualities [7]. What is new is the volume of trade in goods and services, which has reached unprecedented levels over the past century; and the global scale at which trade now occurs. Also, the pattern of trade has morphed into an unequal playing field, where international trade rules tend to benefit disproportionately high-income countries [8-11]. The rise in global production chains, liberalization of global financial flows and stark inequalities in countries' political and bargaining power are at the heart of many of the contentions concerning contemporary global trade.

Health concerns associated with trade have been a feature of national and global policy debate since the establishment of the World Trade Organization (WTO) in 1995 and its extensive suite of trade treaties aimed at progressively liberalizing the cross border flow of goods, services and finance. Such concerns are far from new. Disease has long followed trade routes, from infectious pandemics of past eras to SARS in more recent times. The link between trade and infectious disease has been well documented [12-14]; and there is now an emerging evidence base that global trade is also linked with the rise of chronic disease in many LMICs. This linkage is associated, in part, with the global diffusion of unhealthy lifestyles and health damaging products [15], posing a particular challenge to countries still facing high burdens of communicable disease.

The existing literature on trade and chronic disease has tended to focus on certain health problems, such as diabetes and overnutrition [16,17]. Lacking is an understanding of how such trade affects chronic disease more generally and through multiple pathways. To address this knowledge gap, we developed a generic framework which depicts the determinants and pathways connecting global trade with chronic disease. We then applied this framework to three key risk factors for chronic disease: unhealthy diets, alcohol, and tobacco. This led to specific 'product pathways', which we propose can be further refined and used by health policy-makers to engage with their country's trade policy-makers around health impacts of ongoing trade treaty negotiations, and by researchers to continue refining an evidence base on how global trade is affecting patterns of chronic disease. We focused our evidence gathering primarily on Latin America, sub Saharan Africa, and Asia, where the impact of international trade agreements in the global flow of these products has been subject of greatest health comment and concern.

### Trade, chronicity and chronic disease

'Chronicity' has been proposed as an appropriate lens to address the complexities associated with rising burden of chronic diseases [18] and has been identified as the theme for this special issue. The concept of chronicity has conventionally been applied to understanding the nature of care of chronic diseases [19]. However, the term is also applicable to the *causes *of chronic diseases. Specifically, we view chronicity in two ways: first, as the post-1980s reconfiguration of globalization (particularly economic aspects of trade and investment liberalization following what has been characterized as neo-liberal economic principles)[20], which has led to the international transmission of risk factors for non-communicable disease; and second, as the durability of this model even in the face of multiple, and more recently global, financial crises. Trade-related global market integration has essentially made such disease risk factors 'communicable' (with food, tobacco and alcohol consumption serving as 'vectors'), blurring the conventional distinction between communicable and chronic diseases.

#### Policy space, policy capacity, trade treaty rules, and risks of chronic disease

'Policy space' is the term frequently used to describe "the freedom, scope, and mechanisms that governments have to choose, design and implement public policies to fulfill their aims" [[21], p.7]. Policy capacity refers to the fiscal ability of states to enact those policies or regulations, which depends upon their ability to capture sufficient revenue through taxation for this purpose. Both space and capacity can be affected by trade treaties. One concern with trade treaties is their 'behind the border' shrinking of policy space by prohibiting a range of 'trade-related' domestic regulatory options that could be used to promote healthy habits or, conversely, to restrict unhealthy ones. Although governments still retain substantial policy flexibilities within existing trade treaties, these flexibilities continue to be eroded through ongoing treaty negotiations, notably those associated with bilateral or regional trade treaties. Such treaties are exempt from the *most favoured nation *rule of WTO agreements under which trade terms between any WTO member nations must be given to all member nations. The exemption for bilateral and regional treaties allows for more favourable terms (usually with respect to market access) for countries that participate in them. Regional treaties hold the prospect for more equitable forms of trade amongst countries of similar size or development level [22]. However, such treaties, especially those with wealthier countries or trading blocs, such as the USA and EU, are often 'WTO+.' They include trade, services and finance liberalization commitments, protection of intellectual property rights and agreements on government procurement that go beyond those present in existing WTO trade treaties, and which can limit policy space to a much greater extent than WTO trade rules [22-24].

The primary purpose of all trade treaties is to reduce barriers to cross-border trade. One of the key principles underlying this purpose is *non-discrimination*: foreign goods or committed services covered by a trade treaty must be treated the same as the identical or 'like' domestic good or service. Another principle is *national treatment*. Internal tax and regulatory measures must be applied equally to imported and domestic goods or committed (scheduled) services in order to avoid trade disputes. To protect population health found to be in violation of trade agreements (the so-called *health defense*), governments have to prove that these policies are 'necessary.' Past and ongoing disputes over regulations governing tobacco imports and additives, and alcohol products, highlight the stringency with which this requirement is pursued [25]. Further limitations on the health defense include requirements that domestic regulations that could discriminate against foreign imports, even if treated no differently than national goods, must be based upon international standards or scientific risk assessments [7]. These trade principles constrain policy space. Policy capacity, in turn, refers to the resources states have to monitor or enforce regulations that they are able to promulgate. The issue of capacity is of considerable importance to LMICs, many of which have excellent laws 'on the books' but lack effective enforcement measures. The policy capacity trade issue is that liberalization requires progressive reductions in tariffs (border taxes). Developing countries rely more heavily upon tariffs for their tax revenue than do developed nations. Although developing countries are granted more latitude in retaining higher tariff levels, they are under considerable trade negotiation pressure to lock in and reduce their tariffs, in both multilateral WTO negotiations and notably in bilateral and regional trade treaties. In theory, developing country governments should be able to shift their tax bases from tariffs to sales or income taxes, assuming their economies grow with increased liberalization. In reality, many developing, and most low-income, countries subject to tariff reductions as conditions for loans from the international financial institutions (the World Bank and IMF) have been unable to do so [26,27], partly as a result of inadequate institutions to implement alternate tax regimes [28]. For a majority of these countries there has been a net decline in overall public revenues [29] - a loss in policy capacity - with implications for spending in health, education or public regulations that can affect primary and secondary prevention of chronic disease.

### Generic framework

**Figure 1:**
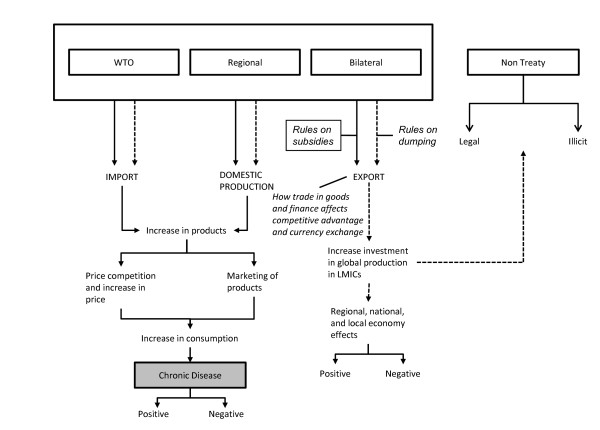
**A generic framework of the linkages between chronic disease and international trade**.

Figure 1 provides a generic framework of the linkages between chronic disease and international trade. Trade can be broadly segmented into two categories: treaty, which includes bilateral, regional or multilateral under the World Trade Organization (WTO) and non-treaty, which includes both legal (but non-treaty) and illicit trade. Trade treaties can affect trade in goods in two main ways: increased trade in raw or finished products (depicted with solid arrow lines) and increased foreign investment in domestic production, manufacturing, and distribution (depicted with dotted arrow lines). Increased imports and domestic production result in increased domestic availability of a particular product. Greater quantity and availability, in turn, increases price competition (lower prices) and marketing and (generally) promotion of the product, both of which lead to increased product consumption. Increased consumption can have positive or negative consequences on chronic diseases depending on whether it is a health-promoting (e.g. nutritious food) or health-damaging (e.g. highly processed food) product. Increased foreign investment in a particular product can also lead to economic growth which, if adequately taxed, can contribute to revenues for health and other health-promoting social programs. However, if this product has harmful effects (e.g. tobacco) increased consumption is more likely to lead to poorer health outcomes, burdening health systems and offsetting any economic gains. Moreover, increased imports and foreign investment can displace domestic producers and manufacturers, which can reduce local revenues, food security (if local food crops are displaced) and increase dependency on foreign companies, making it more difficult to introduce regulations constraining their market growth or raising corporate taxes. Non-treaty trade in products has similar effects apart from legally binding constraints on a country's tariffs or domestic policies. Illicit trade is difficult to document for most products and therefore we do not discuss it in this paper.

### Specific pathway products

#### Food trade and chronic disease

The pathways linking trade and foreign direct investment from food to chronic disease are described below. We identify three general pathways which relate to the changes in the food system: growth of transnational food corporations; liberalization of international food trade and investment and global food advertising and promotion. While international trade is a key driver for rising chronic disease linked to changing food consumption patterns, there are other factors, such as urbanization, which are also important [30,31], but a discussion of these factors are beyond the scope of this paper. Urbanization, however, can also be indirectly linked to international trade, becoming part of the broader social system that we discuss briefly [32].

##### Growth of transnational food corporations (TFCs)

Food production, distribution, and retailing have been consolidated into a small number of transnational food corporations (TFCs). Food retailers in particular have undergone an intense and rapid transformation; changes that occurred in regions such as Latin America between 1990 and 2000 took place in the US over a period of 50 years [33]. In 2003, the top 30 food retailers controlled almost 30% of the market in Latin America and 19% in Asia and Oceania [32]. Reardon and his colleagues have labeled the retail transformation beginning in the early 1990s as a 'take-off' period [34], launching a 'supermarket revolution' and the rapid spread of fast food chains. Supermarkets are rapidly increasing (they are now the dominant food retailer in Latin America), and the transformation can be characterized in two ways [33]. First, rapid consolidation occurred where a small number of supermarket chains usurped domestic chains. The second way in which this transformation is occurring is through rapid multinationalization. For example, Wal-Mart has emerged as the largest chain since it began to sell food 10 years ago to the extent that Mexicans spend 3 out of every 10 pesos on food at Wal-Mart [35].

The growth of supermarkets during the 1990s can be attributed to demand side factors, notably urbanization, the entry of women into the workforce, and economic growth [33]. The supply side was driven by trade liberalization and foreign direct investment (FDI). Conditions for FDI were facilitated through the easing on FDI regulations as part of structural adjustment programs and free trade agreements. FDI has played a critical role in the diet transition as it has especially targeted highly processed foods [36]. There is a close correspondence between a rise in FDI and increased investments in processed foods. In Latin America, between 1988 and 1997, FDI in food industries grew from US$ 222 million to US$ 3.3 billion [37]. Supermarkets have focused on highly processed foods because of their long shelf lives and for the potential economies of scale [38]. There is a strong plausible link between the rise of supermarkets and dietary changes, although there is little empirical evidence due to a simple lack of studies on this topic [32].

##### Liberalization of international food trade and investment

Liberalization of trade - eliminating quotas, reducing tariffs, and privatizing state trade agencies - was adopted by many LMICs either voluntarily or as a condition of structural adjustment loans from the international financial institutions initiated in the 1980s, with a quickening pace during the 1990s as many countries entered into global, regional, and bilateral trade agreements [32]. Food was first represented in multilateral trade treaties with the formation of the WTO in 1995 and adoption of the Agreement on Agriculture (AoA). Before this time, agricultural trade existed largely outside of formal trade treaties, and developing countries did not have to reciprocate in granting greater market access to developed country exports. With the WTO's trade rules and dispute settlement procedures, developing countries are under increasing obligation and ongoing negotiation pressures to lower tariffs, export subsidies and domestic agriculture support (AoA), as well as to open themselves to FDI in food-related sectors they may have committed under the General Agreement on Trade in Services (GATS). Alongside a growing number of bilateral and regional treaties, such as the North American Free Trade Agreement (NAFTA), the Central American Free Trade Agreement (CAFTA), and the Southern Common Market (MERCOSUR), regulation of international food trade and investment is increasingly governed by trade treaty rules. A specific example of trade treaty effects on health-related food policies includes the long-standing dispute between the European Union and several countries over the EU ban on hormone-treated beef (the ban violates requirements for scientific risk assessments under the WTO Agreement on Sanitary and Phytosanitary Standards) [39]. Another example involves the threat of a trade dispute involving the Gerber company and Guatemala over the latter's effort to abide by the infant formula code (International Code of Marketing of Breast-milk Substitutes) by banning the 'pudgy baby' picture on Gerber infant formulas (which the company argued was an infringement of its intellectual property rights) [39].

At the start of the new millennium, food represented 11% of international trade (likely more today), with the rise of processed food occurring more quickly than primary agricultural products [40]. While international trade of food and food-products has increased, so have the level of subsidies provided to agricultural producers in high-income countries (notably the USA, the EU and Japan) with much of their produce (particularly American and European) going to export markets. This has led some trade policy analysts to argue that the high level of subsides can be viewed as dumping [41], defined in trade terms as goods entering a foreign market at less than 'normal' prices. These subsidies are due to be reduced under the terms of the AoA (which gave WTO member nations a 10 year moratorium from trade disputes related to agriculture, which expired on December 31, 2004); although both the US and the EU have been altering slightly the terms of their subsidies to allow them to still qualify under the AoA's complex set of 'boxes' permitting some, but disallowing other, supports to domestic producers. Much prevailing criticism of subsidies is that they damage the value of food exports from developing countries by suppressing world prices. From a public health vantage, eliminating production subsidies on unhealthy food products (such as fats and sugars) is likely to do more health good than harm for all countries. But their elimination on healthier and essential food products could do more harm than good to many low-income countries which have become net-food importers - as a result of population growth, loss of arable land and years of advice to shift from food products for domestic consumption to non-food cash crops (cotton, coffee, tobacco) for export [29,42].

FDI in food-related production, processing and retailing, enhanced by reducing investment barriers, has increased the presence of TFCs in most developing countries. This presence can increase food availability through reduction in retail prices following the removal of import barriers on food, depending on the dynamics of international and domestic prices. Food retail prices can also be lowered by the reduction of investment barriers since TFCs often purchase agricultural products at lower cost and promote economies of scale, but they also benefit from the lower agricultural cost of their own products. Hawkes and Thow demonstrate these effects in their analysis of the Central America - Dominican Republic - Free Trade Agreement [43], which the authors argue will likely lead to greater consumption of highly processed food, meat, and other non-traditional foods in Central America.

Liberalization of trade in food products can increase availability and lower retail prices [43,44]. Food availability increases due to reductions of import barriers on foods; although total food availability depends on whether or not there is a concomitant decline in domestic production, or the amount of domestic production that converts to export crops. Impacts on domestic production raise concerns about short- and longer-term food security. A recent study by the United Nations Food and Agriculture Organization (FAO) examined trade liberalization and food security in fifteen small and large developing countries (Chile, Guatemala, Guyana, Peru, Cameroon, Ghana, Kenya, Malawi, Morocco, Nigeria, Senegal, Tanzania, Uganda, China, and India). Their key finding was that "trade reform can be damaging to food security in the short to medium term if it is introduced without a policy package designed to offset the negative effects of liberalization" [[42], p. 75). The study went on to caution that trade reforms generally benefit farmers producing exports crops, but have negative impacts on farmers producing import-competing food stuffs, especially those that are highly subsidized by exporting countries. For low-income countries whose economies are still heavily dependent on agriculture, raising agricultural productivity and creating non-agricultural employment should precede trade reforms such as tariffs reductions on crops grown by low-income households. Production subsidies in developing countries, the report concluded, should also be permitted if these are directed principally to subsistence or resource-poor farmers.

Trade liberalization can also affect food security at the household level. Studies by the International Food Policy Research Institute (IFPRI) in the 1980s examined the nutritional impact of a series of cash cropping schemes in ten developing countries. The findings suggested that cash cropping generally results in higher incomes and spending on food, but has a relatively small impact on energy intake, and, in most cases, little or no impact on childhood malnutrition [45]. Several projects actually had negative impacts on nutrition. Where improvements did occur, most were attributed to the control of income within the household. Female-controlled incomes were related to higher levels of caloric intakes among children, as women are more likely than men to allocate resources towards food.

Hawkes and her colleagues reviewed the available evidence on the links between international trade and dietary patterns [32]. They found supporting evidence, notably from India and the Pacific Islands, that the increase in international trade has shifted dietary patterns from local, 'healthy' diets to the consumption of fattier diets. One study from Colombia found that the proportion of calories consumed from imported foods has increased over time, but the extent to its contribution to increased energy availability is not clear. There was also some limited evidence to support some authors' claims that changing diets has influenced trade and that international trade is simply responding to new demands.

Food exports are another core component of the liberalization of the international food trade [46]. Support for export industries is promoted by the International Trade Centre, which is a cooperative agency composed of the United Nations Conference on Trade and Development and the WTO. The focus of these policies has been especially on the exportation of 'cash crops'. Increasing cash crops decreases land available for domestic crops, requires fewer farmers for domestic production, and reduces the production of traditional food crops for local diets. This has led to a decline in consumption of traditional food crops and often a decline in the 'prestige' of traditional foods. These effects are particularly harmful in areas where undernutrition rates are still high and influence levels of food security for poor and marginalized groups. More recently, several high-income countries have been entering into long-term land lease arrangements with poorer, indebted countries to grow food specifically to meet the needs of citizens of the high-income nations. This new development has increased concern over future food security in poorer countries [47-49].

##### Global food advertising and promotion

Advertising and promotion marks the third pathway through which trade is affecting food systems and chronic disease. In order to dominate in competitive food retailing markets, corporations employ aggressive marketing techniques. Spending on food advertising is now higher than it is for tobacco [35]. In 2004, Coca Cola spent US$ 2.2 billion and PepsiCo spent US$ 1.7 billion on marketing of soft drinks [37]. The global food advertising has been steadily growing and the advertisement market is controlled by a few communications networks [32]. Processed food, especially targeted to children, has been the main focus of promotion and advertising [32]. FDI has also played a major role in marketing products [25,35]. Global food advertising has especially targeted developing countries in its search for new markets, with a focus on highly processed foods. In 2002, almost 60% of food advertisements in Brazil were for foods high in fats and sweeteners [50].

Advertising and product marketing has contributed to changing cultural expectations of food [37] and the "systematic molding of taste by giant corporations" [[35], p. 1559]. Marketing has been especially targeted to youth. During the late 1990s, soft-drink companies targeted school children by selling products in attractive combination packages in schools in Mexico and Colombia, which led to a 50% increase in soft drink sales among children [32]. Evidence from industrialized and developing countries found that children engage with food advertising and that there is clear link between advertising to children and the consumption of these products [51,52].

##### Social change

Given that the diet transition advances with rising incomes, urbanization, and changes in the labour market, it can be expected that trade liberalization- when it leads to economic growth, increasing employment, and urbanization - will also influence the diet transition as changes in lifestyles and new food demands arise [32]. However, since liberalization does not always or necessarily lead to economic growth, diets may not be markedly influenced. When trade-related economic growth does occur, economic inequalities that generally accompany such growth can lead to the poorest groups with limited access to food and the abilityto meet their basic nutritional requirements. This can generate patterns of overnutrition and undernutrition in the same country, particularly in LMICs. There are only a few studies that have demonstrated links between trade liberalization, employment conditions, and nutrition. Better jobs and higher incomes can lead to changes in food preferences and capacity to buy new foods. However, time constraints and more women in the labour force can lead to increased consumption of energy-dense, time saving foods. Job insecurities and limited access to family and welfare benefits can also restrict healthy food choices. In sum, although there are links between economic growth, urbanization, and changing labour markets, the nature of these links are not clear and likely depend on the particular context.

#### Tobacco trade and health

Trade liberalization in tobacco products is a concern for its potential to offset declining use in developed countries by penetrating new markets in developing nations. Trade can increase the disease consequences of tobacco consumption through two main pathways: trade and investment liberalization; and the impact of trade rules on government policy space.

##### Liberalization of international tobacco trade and investment

Trade liberalization has led to increased tobacco consumption in LMICs [53] through a combination of tariffs reduction, liberalization in FDI and minimal national tobacco control measures. This combination of factors increases competition in domestic markets, contributes to a reduction in the prices of tobacco products and an increase in advertising and promotion expenditures; all of which lead to increases in tobacco consumption. As one example of this, Honjo and Kawachifound that market liberalization lead to a one year increase in US tobacco products in Japan from 16% in 1986 to 32% in 1987 and a corresponding stall in the decline of tobacco consumption among adults and increase in the level of consumption among adolescent girls [54]. When South Korea opened its domestic market to US tobacco company cigarette imports there was an 11% increase in smoking among males and an 8% increase among females in just one year [55]. Similar interactions have taken place in bilateral trade agreements, including an agreement between the US and China in which China was required to cut tariffs on imported cigarettes. Consumption patterns corresponded with the abolition of tariffs, expanding sales networks and the removal of advertising and marketing restrictions, all policy strategies explicitly pursued by tobacco transnational companies to increase LMIC consumption rates [56]. McGrady further cautions that 'the provisions of trade agreements governing non-tariff barriers to trade will limit effective and comprehensive tobacco control' [57].

While using trade treaties to lower tobacco tariffs has been one strategy adopted by tobacco companies to increase LMIC consumption, an arguably more critical strategy has involved using financial market liberalization to control domestic tobacco industries worldwide. Referring to a now famous GATT dispute in 1990 involving Thailand and the United States, Callard and colleagues (2001) speculate that transnational tobacco companies (TTCs) sought to buy out or enter into a joint venture with the Thai government's tobacco monopoly in order to enhance their economic foothold in a large market and increase their political influence with the goal of weakening tobacco control legislation [58]. GATS mode 3 (commercial presence) facilitates such investment when countries have committed different facets of their domestic tobacco industry to liberalization, although the explosive growth in bilateral investment treaties likely play an even greater role. Philip Morris, an American TTC, draws over half of its cigarette profits from overseas [59]. Less than ten years ago it was estimated that British American Tobacco controlled 50% of all Latin American cigarette sales [56]. In the Dominican Republic, Philip Morris became sole owner of cigarette division *Industria de Tabaco León Jimenes SA *and as a report of this buy-out suggests:

Philip Morris could benefit and increase its market share in the Dominican Republic through more aggressive marketing now that it has complete control over the cigarette division. Philip Morris also could benefit from DR-CAFTA (Central American Free Trade Agreement) by exporting the products it manufactures in the Dominican Republic to Central America [60].

A World Bank study estimated that cigarette production in LMICs rose from 40 to 70% in the past few decades [61], the result primarily of the movement of TTCs into such countries through domestic company acquisition and foreign direct investment. In Argentina, for example, approximately 90% of the tobacco market is now controlled by two tobacco corporations (Philip Morris Corporation and British American Tobacco) neither being domestically owned [50]. In South Africa, British American Tobacco owns 94% of the tobacco market [62]. Foreign investment, in turn, is associated with increased consumption: Gilmore and McKee found that, amongst former Soviet Union republics, those countries that received foreign direct investment from TTCs between 1991 and 2001 saw an increase in tobacco consumption of 51% compared to a 3% drop in those that did not [63].

Between 1970 and 2000 the number of hectares devoted to tobacco growing more than doubled in countries such as Honduras, Guatemala, Uruguay and Haiti [64]. In Brazil, the amount of land committed to tobacco cultivation increased by approximately 60000 hectares [65]. This increase corresponds with the rapid opening of previously closed markets, the increased push for trade liberalization and the growth of the TTCs [66]. While some tobacco farmers and producers may benefit from this shift to tobacco crop production (often for export as well as for domestic purposes), this shift has potential negative implications for domestic food security and access to nutritional foods with consequent risks to health, especially for the poor. It also poses direct health risks, especially to children who are frequently involved in tobacco harvest in low-income countries, where most of the world's tobacco is now cultivated [67]. A recent study of child tobacco workers in Malawi, the fifth leading tobacco producer, estimates that 78,000 children are exposed to 'Green Tobacco Sickness,' absorbing nicotine at rates equivalent to smoking up to 50 cigarettes a day [68].

##### Trade rules and government policy space

Tobacco products generally fall under the WTO's General Agreement on Tariffs and Trade (GATT), concerned primarily with the reduction of import taxes; and the Agreement on Technical Barriers to Trade, which covers non-tariff barriers to trade [53]. Tobacco production is also governed by the AoA with respect to permissible vs. non-prohibited subsidies to tobacco farmers. Tobacco marketing is covered by both the GATS, with respect to advertising, and TRIPS, with respect to regulatory restrictions that might encroach on cigarette logos as 'intellectual property rights'. The WTO system makes tacit reference to health as an interpretative principle [69]; and there are explicit exceptions that allow countries to avoid trade rule compliance if it is 'necessary to protect human, animal or plant life and health' (GATT article XX(b); GATS XIV(b)). Dispute panels, however, have generally applied a stringent necessity test to these exceptions, requiring, in regard to tobacco control, that countries provide sufficient evidence that particular health measures such as labeling restrictions on cigarette packages are essential to protect the health of the population, and that there is no other 'least trade restrictive' option available.

Trade treaties enable tobacco and tobacco products to cross borders more easily. TTCs, in turn, have sought to increase their share of the domestic market in LMICs through strategies that enhance the social image of smoking [56] such as distributing cigarettes to youth, public advertising and lobbying governments to ensure that such strategies are not countered by legislation [70]. Although trade negotiations have been used by TTCs as opportunities to ensure that domestic regulations do not seriously imperil such strategies [71], the Framework Convention on Tobacco Control (FCTC), negotiated under the WHO system, seeks to strengthen through a global agreement tobacco control policies to be pursued by all WHO member states. The FCTC does show some promise for providing an international legal basis for health protection over trade and foreign investment - that is, maintaining or enhancing national policy space. However, Lo argues that unless guidelines are specified in the FCTC to restrict the foreign direct investment of the tobacco industry (which is not presently the case) [72], the industry can continue to avoid tariff barriers (finished goods) while still increasing their presence in domestic markets.

The FCTC contains specific provisions that, assuming foreign tobacco products are treated the same as domestic ones (the non-discrimination standard of the WTO), a country's tobacco control measures should not be subject to a trade dispute. For example Article 11 of the FCTC makes the explicit provision that warning labels on cigarette packages must be "50% or more of the principal display areas" with 30% as an absolute minimum [[73], p. 10]. A government can introduce, without consequence from trade regimes, this provision as long as the legislation does not discriminate between international and domestic cigarette packaging. Tobacco control measures that exceed the minimum standards set forth by the FCTC, however, may be challenged (and are being challenged) under both the WTO system and bilateral investment treaties.

Further challenges arise under bilateral investment treaties, which permit private companies to directly sue national governments for perceived expropriation of their property and earnings (real or potential). In a recent case, Philip Morris challenged Uruguay's decision to implement larger warning labels on tobacco packages than the minimum referenced in the FCTC. It used rules set out in a Swiss-Uruguay investment treaty, arguing that such warning labels violated its intellectual property rights by reducing the space in which it could feature its 'brand' name and logos [74]. The difficulty with disputes involving intellectual property rights (whether under the TRIPS agreement or bilateral or regional 'TRIPS-plus' treaties) is that the specific trade rules covering such protection remain ambiguous and difficult to interpret [75].

Moreover, as Bollyky and Gostin point out, "nearly every investment and trade agreement negotiated by the United States eliminates or reduces trading partners’ tobacco tariffs and protects US tobacco companies' overseas manufacturing and investment”  [[76], p.2637]. The USA remains one of the few countries not to ratify the FCTC, and devotes less than 0.1% of its global health budget to global tobacco control. The lack of US support for tough international tobacco control initiatives may be why enforceable and profitable trade rules continue to exert more force than normative and unenforceable public health treaties.

#### Alcohol trade and chronic disease

Concerns are also rising about the impact of numerous WTO agreements on liberalized trade in alcohol and consequent alcohol-related health problems. Below we discuss four pathways linking trade and investment liberalization to alcohol-related chronic diseases: increased availability, affordability, and marketing of alcohol; decreased alcohol control policies; domestic health-related economic effects and non-treaty trade in alcohol.

##### Increase availability, affordability, and marketing of alcohol

The production, distribution, and marketing of alcohol are becoming increasingly globalized. Most alcoholic beverages are largely purchased in the country of production, although cross-border trade in spirits (primarily those produced in high-income countries) has become subject to disputes over differential tax regimes (primarily exercised by LMICs), a point addressed later. More importantly, and as with tobacco, international alcohol brands are now being produced industrially in plants owned, co-owned or licensed by multinational corporations [77]. The penetration of transnational alcohol corporations in LMIC markets has increased the availability, affordability, and marketing of alcohol products [78,79] all of which affect consumption rates.

With other factors held constant, a rise in alcohol prices leads to a reduction in the consumption of alcohol and alcohol-related harms [80]. Public health benefits result from higher alcohol prices, even though demand for alcohol is relatively inelastic to price [81]. A rise in prices will generally lead to a reduction in consumption that is smaller as a percentage compared to that of the price increase. Increasing prices tend to have a greater impact over the long term rather than in the short term. In addition, young drinkers and frequent and heavier drinkers (two groups for whom the health risks of consumption are generally greater) are more likely to reduce their consumption compared to older drinkers and infrequent and lighter drinkers [80,82].

Greater diversity of alcohol products made available through reduced tariffs on imports can increase overall alcohol consumption as these products can target a variety of tastes and preferences, although in some cases consumers may simply shift from domestic to foreign products [83]. Also, many of the new foreign beverages contain higher alcohol content compared to domestic products [78,84].

As alcohol companies 'thirst for new markets' [85], intensive marketing practices are adopted as a means to increase consumption of alcohol, particularly in LMICs [83]. The role of advertising is a critical factor in differentiating between 'globalised' and other types of alcohol [79]. Whereas traditional local alcoholic products were marketed based on availability, quality, and price, a global alcohol product is "synonymous with its imagery...represents a culture of its own" [[77], p. S471]. Alcohol is being marketed through increasingly sophisticated avenues, including direct marketing (e.g. podcasting, cell phones), mainstream media, and via sporting and cultural events. Researchers have demonstrated that advertising is associated with alcohol use by youths, notably initiation of drinking and hazardous drinking patterns [80]. 'False advertising', such as marketing products as containing low alcohol when it is consumed as a mixed drink or the targeting of vulnerable groups have been employed as a means of counteracting health trends by consumers towards non alcoholic beverages or drinks with lower alcohol content [83].

The EU and the USA in current WTO-GATS negotiations are aggressively pursuing unlimited liberalization commitments in advertising; and "the World Spirits Alliance has described the Doha Round as offering 'an excellent opportunity for the international distilled spirits industry to create new opportunities to expand its exports to world markets,'" identifying "liberalisation of restrictions on services, including distribution and advertising'' as one of its top five priorities for the new trade round [[86], p.367].

##### Decrease alcohol control policies

In the context of trade negotiations, alcohol can be treated as a 'commercial good' to be freely traded as any other good. The health-damaging properties of alcohol have been largely ignored. While there are also some health benefits related to modest alcohol consumption [87], there are major health risks which are generally confined to alcoholism, impaired driving, injuries, and fetal alcohol syndrome, although even moderate alcohol use carries some health risk. Rhem and colleagues estimate that 3.8% of all global deaths and 4.6% of global disability-adjusted life-years are attributable to alcohol [88]. Domestic regulators must ensure that their alcohol policies comply with conditions set out in trade treaties, potentially reducing their capacity to implement appropriate policies. Many of the policies that can help reduce alcohol-related harm (e.g. tariffs, taxes, licensing, labeling, regulation of the size of alcoholic beverage containers, identifying certain brands as 'noxious' or 'injurious') are considered to be barriers to trade under several WTO trade agreements [83].

Reducing the control of state monopolies and enterprises is a key element of many trade treaties. Researchers have observed an increase in alcohol consumption and alcohol-related problems following the elimination of government control of alcohol measures. The Nordic countries are a case in point. Since the early 20^th ^century, Finland, Norway, and Sweden had state monopolies on production and wholesale, import and export, and off-premise retail monopolies - all with the overarching goal of reducing individual and social harm from alcohol consumption [89]. Following integration into the European Union (EU) and the European Economic Area (EEA), an 1994 agreement for a single European market (Norway is not a member of the EU, but entered into the EEA), these countries have had to yield to pressure to undertake trade activities that adopt the principles of national treatment or non-discrimination. Alavaikko and Österberg demonstrated that following Finland's entry into the European Union in 1995, the country's markets opened and the state alcohol monopoly company [90], Alko, lost its traditional capacity for alcohol decision-making policy. Mäkelä and Österberg observed that alcohol consumption increased 10% in 2004 and levels have remained higher ever since [91].

Another key element of trade treaties is a greater 'harmonization' of taxes and duties on alcoholic beverages [78]. In particular, national alcohol taxation systems have been directly affected by the application of the 'national treatment' clause. Recently, the EU has requested the WTO to examine the Philippine's excise tax regime, which includes a higher tax rate on imported spirits than domestic spirits, which are taxed at a flat rate [92]. The EU claims that this provides unfair market competition, whereas the Philippines defends the law on the grounds that it provides support to indigenous communities, producing spirits from their raw materials, like coconut and sugarcane.

Countries have succeeded in maintaining alcohol control policies when they have been able to demonstrate that the law was protective to the health of the population; exceptions for such a purpose exist in both the GATT and in the GATS. When health arguments are not specifically invoked, it is unlikely that a country will win a dispute. Chile, for example, lost their case on defending their tax policy on imported spirits before the WTO [83]. Chile levied a disproportionately high tax rate on spirits that had alcohol content higher than 40 percent. It did not invoke public health arguments, instead relying on the argument that its policy was non-discriminatory, since it applied to all alcohol products, both domestic and imported. The EU, in this dispute, countered that most varieties of *pisco*, the domestically produced spirit, by law was required to have an alcohol content below 35 percent; whereas most imported spirits had alcohol content of 40 percent or above; thus having the effect of providing unfair tax advantage to the domestic product. The WTO agreed, ruling in favour of the EU. In its ruling it noted that "members of the WTO are free to tax distilled alcoholic beverages on the basis of their alcohol content and price," which would appear to allow for a health argument to be made against high alcohol content imports. But such a policy would only be permissible "as long as the tax classification is not applied so as to protect domestic production over imports," meaning that a discriminatory tax on alcohol content, even if designed for public health purposes, could be found in violation of trade treaty obligations [93].

There have been countries that have won cases on the basis of a health defense. One example is France's *Loi Evin*, implemented to restrict alcohol advertising [86]. The European Court which heard this case (which applied intra-European trade rules) found that while these prohibitions conflicted with the European Treaty (Article 59, which stipulates abolishing restrictions on the provisions of services, including advertising), the French regulations were deemed appropriate to protecting public health. While European Union law may be more 'health friendly' than WTO trade treaties, Baumberg and Anderson argue that policies motivated purely by health interests may have more flexibility in trade policy than what is often perceived [94]. They call for countries implementing alcohol-restricting policies to pay closer attention to case law in Europe to better understand how to craft alcohol control policies, and to avoid narrowing their policy space during ongoing trade negotiations.

##### Domestic health-related economic impacts

It has been argued that foreign investments by alcohol corporations can 'offset' the harm caused by increased alcohol consumption in LMICs due to potential economic benefits. These benefits include employment and income generation, increased government revenue for governments, a stronger economy through exports and import substitution, and the transfer of technology and skills via multinational corporations [77,89]. However, while global markets can increase employment and promote the transfer of technological advances from high income to LMICs, global trade tends to benefit rich countries - particularly a few global corporations [89]. Employment benefits depend on the local context and the alcohol product. Trade-related growth in foreign private distributors and retailers over local monopolies, for example, can drive out alcohol profits from the local economy [78]. Foreign companies may displace local employment, since their breweries and production facilities often require imported technology [77]. Operation of these facilities tends to require fewer, highly skilled workers. Companies will often bring in expatriates, reducing employment opportunities for local populations who have traditionally worked in the production and trade of alcohol, such as female heads of households. Local populations may be marginalized from participation in this new industry development and unable to reap any benefits in employment or skill development. Foreign corporations can also influence the larger political and economic contexts; as their share of the market increases, so does their power as actors on the national and sub-national scales [79].

High taxes on alcohol can be a positive public finance instrument with public health benefits. However, in order to collect such revenue, countries need effective control over the alcohol supply, which many developing countries do not have [83] and which trade treaty restrictions on differential taxation by alcohol content level weaken. Export-oriented policies for alcohol may not be effective in LMICs, since the global trade necessitates high quality alcohol that can travel long-distances. Few LMICs are able to produce this type of alcohol or to compete against well-established international brands, although tequila and rum are two notable exceptions. In sum, any potential role for global alcohol trade in domestic economic development (with implied trickle-down health benefits) remains ambiguous at best. To address the growing concerns between the links between international trade and alcohol, a Framework Convention on Alcohol Control (FCAC), is being proposed (a point we return to in the conclusion).

#### Trade liberalization, inequity and chronic disease: indirect pathways

Our framework and this article has focused on specific (and what we claim now represent progressive effects of international trade) product pathways. But there are also generalized features of trade and financial market liberalization that have important bearing on chronic disease risks. These features refer primarily to the inequitable impacts of global liberalization on socioeconomic and labour market inequities. Although there is general consensus that extreme poverty globally has fallen in recent decades [95,96], its attribution to trade liberalization remains weak [97] and disproportionate to the quadrupling in global economic product over the same time period. Over 3.2 billion people still live below the World Bank $2/day poverty level, with each unit of global economic growth contributing less than half to poverty reduction today than it did in the 1970s [98]. Trade liberalization is also associated with increased income inequalities within and between countries [99] along with geospatial inequalities in developing countries arising from coastal locations of export-oriented manufacturing (including export-processing zones). A possible reduction in gender income inequalities due to increased women's employment has been observed, but with increased health risks due to unsafe or unhealthy working conditions [100,101], as well as a growing educational-based income and job security divide between 'skilled' and 'unskilled' workers. All forms of stratification can lead to social exclusion (both economic and psychosocial) posing particular health risks for both infectious and chronic disease.

The trade-related risk for chronic disease is most pronounced with respect to economic insecurities and labour market changes. The weight of existing evidence supports the view that trade liberalization increases economic insecurity [102]. Workers and producers in the sectors protected from foreign competition may see revenues decrease or employment disappear when tariffs or regulatory barriers are removed. As full-time manufacturing employment is lost (and not just in high-income, but also in LMICs, see [103]), there are increases in 'non-standard' (insecure, part-time, precarious) forms of employment [104]. There is a close link between economic insecurity and many chronic stress-related diseases such as cardiovascular problems [105]. Insecure employment in particular is associated with increased stress leading to a greater risk of both infectious and chronic disease [106,107].

Trade-related health risks have not gone unnoticed, both specifically and generally. In the case of tobacco trade, the FCTC is in part a response to challenges of a globalized tobacco industry. Its ability to trump trade treaties invoked by TTCs in their pursuit of larger markets is still being tested; although the internationalization of tobacco's singularly negative effects and vilification of the tobacco industry may assist in strengthening the normative, if not narrowly legal, force of the FCTC. There is no alcohol-equivalent to the FCTC, although the WHO recently submitted a draft global strategy to reduce harmful use of alcohol, which included recommendations and proposals for regulating availability, marketing and pricing [108]. There is growing support for the FCAC from diverse actors, including the Indian Government, the American Public Health Association, the World Medical Association, and the WHO Commission on the Social Determinants of Health [109,110]. A FCAC would help to demonstrate that alcohol is not an "ordinary commodity" and help to address global factors influencing its consumption, such as liberalization of marketing [109]. Even if such a Framework Convention were negotiated, problems with its intersection with trade rules (such as those outlined with the FCTC) would likely remain. The fact that the health harms arising from alcohol are more ambiguous may make it more difficult to apply normative pressures under similar treaty dispute situations.

With respect to the generalized issue of trade and chronic disease, there is some evidence that increased social protection programs (e.g. employment insurance, active labour market programs, welfare cash transfers, universal health and education access) can buffer some of the health negative effects of liberalization and global market integration [102,111]. However, excluding a handful of rapidly industrializing middle-income countries, most of the world's developing countries negatively affected by the financial crisis lack the fiscal capacity to expand their social protection programs [112]. Some high income countries affected by the costs of bank bailouts and stimulus spending, or by the recession in the 'real' economy of production and consumption, such as Ireland are making draconian cuts in their existing social protection spending to qualify for IMF loans.

## Conclusion

This article has reviewed extant evidence on the role that trade and financial liberalization has played in increasing the global diffusion of risk factors for chronic disease. The pathways by which trade can affect chronic disease are multiple. These pathways can be direct (increased exposure to harmful or potentially harmful commodities, notably tobacco, obesogenic foods and alcohol) and indirect (through changes in labour markets leading to economic and employment insecurity, associated with increased chronic disease risk). There is some potential for trade treaties to aid in reducing the global diffusion of risk factors, such as enforcing an end to domestic subsidies for agricultural exports harmful to health (e.g., sugars, fats, tobacco) or removal of tariffs on the import of drugs used to treat NCDs. However, as this article has elaborated, there remains considerable actual or potential health-harm in trade treaties when such treaties are driven by liberalization as the policy end and with only minimal regard to the health consequences.

This potential has been noticed in the run-up to the UN Summit on Noncommunicable Diseases taking place in September, 2011. A meeting of African health ministers in early April 2011 issued a declaration on NCDs stating, *inter alia*, that "although globalization, trade and urbanization are important in human development, they are also major external drivers responsible for widening health inequities within and between countries and populations" demanding "the integration of health in all policies across sectors in order to address NCD risk factors and determinants [113]." This declaration repeats a theme woven throughout the WHO's *Global Status Report on Noncommunicable Diseases 2010*, which noted that "the rapidly growing burden of NCDs in developing countries is not only accelerated by population ageing; it is also driven by the negative effects of globalization, for example, unfair trade and irresponsible marketing" [[114], p.33]. WHO Director-General, Margaret Chan, was even more forceful in her comments to the April, 2011 First Global Ministerial Conference on Health Lifestyles and Noncommunicable Disease Control convened in Moscow, regarded as an agenda-setting event for the September UN Summit:

Today, many of the threats to health that contribute to noncommunicable diseases come from corporations that are big, rich and powerful, driven by commercial interests, and far less friendly to health. ... Today, more than half of the world's population lives in an urban setting. Slums need corner food stores that sell fresh produce, not just packaged junk with a cheap price and a long shelf-life [115].

While not referencing trade *per se*, the outcomes Chan cites are logically and empirically linked to trade and the globalized food, tobacco and spirits industries. Yet, notwithstanding the exclusion of the tobacco industry from the Moscow Conference, many of these same globally trading corporations were present to participate in the Conference. Press reports of the Conference quote some of these corporate representatives complaining that companies are "unfairly blamed for consumer's choices" or that "the overfed are voluntarily overfed" [[116], p. 10], reinforcing a concern implicit in the Conference's emphasis on 'healthy lifestyles' that intervention strategies for NCD control could take the easy path of regulating individual health behaviours rather than corporate economic or social practices. Such practices are so far being addressed through calls for voluntary corporate social responsibility, despite (as one example) over 30 years of repeated non-compliance with the voluntary International Code of Marketing of Breastmilk Substitutes. Any reduction in non-compliance with this Code was largely a result of activist groups supporting governments to write Code requirements into their (enforceable) national legislation [117]. Worryingly, the declaration issued by the Moscow Conference makes no reference to globalization, trade or even to EU- and USA-led initiatives in bilateral or regional trade treaties to extend intellectual property rights (IPRs), which could impede access to drugs or diagnostics important to the treatment of NCDs. Indeed, there is little reference to IPRs in any of the expressed concerns about access to medicines in any of the advance commentaries leading up to the September UN Summit.

These lacunae in discussion of key global determinants of chronic disease prevention and treatment are surprising, given the evidence and argument advanced on such determinants issue in recent years. Whether the direct or indirect disease implications of global market integration enters seriously in discussions of global, regional and bilateral trade treaty negotiations remains a moot question. But the same applies to whether trade-related implications of chronic disease prevention and management will enter more forcefully into new global debates and plans to address the rising pandemic of these diseases. This article, in mapping some of what is known of the relationships between the two, hopefully will encourage constructive actions from both sides of the trade/health table. At minimum, we should expect explicit recognition of the globalization and trade-related dimension of the world's rising burden of chronic disease when nations meet to discuss plans of action later this year. Ideally, this should also look for commitments to ensure that trade negotiators take full account of the health impacts of the treaties they develop, with sufficient time and public disclosure of treaty elements for those in the public health community (governmental and civil society) to assess, analyze and respond.

## Competing interests

The authors declare that they have no competing interests.

## Authors' contributions

All authors contributed to the literature reviews and technical report to PAHO from which this paper is adapted. RLab drafted the introductory and concluding sections, KM wrote the sections on alcohol and food trade, RLen wrote the section on tobacco. All authors contributed to revisions of the final manuscript; and read and approved the final manuscript.
